# Core protocol development for phase 2/3 clinical trials in the leukodystrophy vanishing white matter: a consensus statement by the VWM consortium and patient advocates

**DOI:** 10.1186/s12883-023-03354-9

**Published:** 2023-08-17

**Authors:** Daphne H. Schoenmakers, Prisca S. Leferink, Adeline Vanderver, Joshua L. Bonkowsky, Ingeborg Krägeloh-Mann, Geneviève Bernard, Enrico Bertini, Ali Fatemi, Brent L. Fogel, Nicole I. Wolf, Donna Skwirut, Allyson Buck, Brett Holberg, Elise F. Saunier-Vivar, Robert Rauner, Hanka Dekker, Pieter van Bokhoven, Menno D. Stellingwerff, Johannes Berkhof, Marjo S. van der Knaap

**Affiliations:** 1 Department of Child Neurology, Emma’s Children’s Hospital, Amsterdam UMC Location Vrije Universiteit, Amsterdam, The Netherlands; 2 Amsterdam Leukodystrophy Center, Amsterdam Neuroscience, Cellular & Molecular Mechanisms, Amsterdam, The Netherlands; 3 Department of Endocrinology and Metabolism, Platform “Medicine for Society”, Amsterdam UMC Location University of Amsterdam, Amsterdam, The Netherlands; 4 IXA Neuroscience, Amsterdam Neuroscience, Amsterdam UMC Location Vrije Universiteit, Amsterdam, The Netherlands; 5 Division of Neurology, Children’s Hospital of Philadelphia, Philadelphia, USA; 6 Department of Neurology, Perelman School of Medicine, University of Pennsylvania, Philadelphia, USA; 7 Division of Pediatric Neurology, Department of Pediatrics, University of Utah School of Medicine, Salt Lake City, Utah USA; 8 Primary Children’s Hospital, Intermountain Healthcare, Salt Lake City, Utah USA; 9 Department of Developmental and Child Neurology, Social Pediatrics, University Children’s Hospital Tübingen, Tübingen, Germany; 10 Departments of Neurology and Neurosurgery, Pediatrics and Human Genetics, McGill University; Department Specialized Medicine, Division of Medical Genetics, McGill University Health Center, Montreal, Canada; 11 Child Health and Human Development Program, Research Institute of the McGill University Health Center, Montreal, Canada; 12 Research Unit of Neuromuscular and Neurodegenerative Diseases, Translational Pediatrics and Clinical Genetics Research Division, Ospedale Pediatrico Bambino Gesù, IRCCS, Rome, Italy; 13 Kennedy Krieger Institute, Johns Hopkins University, Baltimore, MD USA; 14 Los Angeles David Geffen School of Medicine, University of California, Los Angeles, USA; 15 United Leukodystrophy Foundation, DeKalb Illinois, USA; 16 VWM Families Foundation, Greenwhich, CT USA; 17 Research Department, European Leukodystrophies Association International and European Leukodystrophies Association France, Paris, France; 18 Vereniging Volwassenen, Kinderen en Stofwisselingsziekten, Zwolle, The Netherlands; 19 Department of Epidemiology and Data Science, Amsterdam UMC Location Vrije Universiteit, Amsterdam, The Netherlands; 20 Department of Integrative Neurophysiology, Center for Neurogenomics and Cognitive Research, Vrije Universiteit, Amsterdam, The Netherlands

**Keywords:** Innovative trial design, Trial protocol, Core protocol, Vanishing white matter, Leukodystrophy, Orphan disease

## Abstract

**Background:**

The leukodystrophy “Vanishing White Matter” (VWM) is an orphan disease with neurological decline and high mortality. Currently, VWM has no approved treatments, but advances in understanding pathophysiology have led to identification of promising therapies. Several investigational medicinal products are either in or about to enter clinical trial phase. Clinical trials in VWM pose serious challenges, as VWM has an episodic disease course; disease phenotype is highly heterogeneous and predictable only for early onset; and study power is limited by the small patient numbers. To address these challenges and accelerate therapy delivery, the VWM Consortium, a group of academic clinicians with expertise in VWM, decided to develop a core protocol to function as a template for trials, to improve trial design and facilitate sharing of control data, while permitting flexibility regarding other trial details. Overall aims of the core protocol are to collect safety, tolerability, and efficacy data for treatment assessment and marketing authorization.

**Methods:**

To develop the core protocol, the VWM Consortium designated a committee, including clinician members of the VWM Consortium, family and patient group advocates, and experts in statistics, clinical trial design and alliancing with industries. We drafted three age-specific protocols, to stratify into more homogeneous patient groups, of ages ≥ 18 years, ≥ 6 to < 18 years and < 6 years. We chose double‐blind, randomized, placebo-controlled design for patients aged ≥ 6 years; and open-label non-randomized natural-history-controlled design for patients < 6 years. The protocol describes study populations, age-specific endpoints, inclusion and exclusion criteria, study schedules, sample size determinations, and statistical considerations.

**Discussion:**

The core protocol provides a shared uniformity across trials, enables a pool of shared controls, and reduces the total number of patients necessary per trial, limiting the number of patients on placebo. All VWM clinical trials are suggested to adhere to the core protocol. Other trial components such as choice of primary outcome, pharmacokinetics, pharmacodynamics, and biomarkers are flexible and unconstrained by the core protocol. Each sponsor is responsible for their trial execution, while the control data are handled by a shared research organization. This core protocol benefits the efficiency of parallel and consecutive trials in VWM, and we hope accelerates time to availability of treatments for VWM.

**Trial registration:**

NA. From a scientific and ethical perspective, it is strongly recommended that all interventional trials using this core protocol are registered in a clinical trial register.

## Background

### Vanishing white matter

Vanishing White Matter (VWM, OMIM #603896) is a rare leukodystrophy caused by biallelic pathogenic variants in any of the five genes *EIF2B1-5*, encoding the five subunits of eukaryotic initiation factor 2B (eIF2B). The only known epidemiological data come from the Netherlands, where the incidence is approximately 1:100,000 live births and the prevalence is approximately 1.3:1,000,000 inhabitants [1]. The incidence and prevalence in other countries is likely similar based on studies of genomic databases [2, 3], making it an orphan disease. VWM is characterized by chronic decline with stress-induced episodes of rapid decline, followed by death or partial or complete recovery. Patients develop motor and cognitive disabilities and die after years of progressive handicap [1]. The diagnosis is made when patients present with neurological decline or when family screening is performed because of an affected sibling. An MRI pattern of brain white matter rarefaction prompts genetic testing to confirm the diagnosis [4]. The age at manifestation varies from antenatal period to adulthood. Earlier onset is associated with a more severe phenotype [1, 5].

Two-thirds of the patients have disease onset < 6 years of age; they most often experience rapid neurological decline with a short life expectancy [1]. For patients with onset after 6 years, the disease course is more variable and often more protracted [1]. Life expectancy is shortened, but patients may survive for decades [1]. Because survival is much shorter for patients with onset before 6 years than for patients with later onset, two-third of the living patients is 18 years or older and living patients below 6 years are scarce [3]. In children with VWM, disability is dominated by motor signs (ataxia and spasticity); in adults, the disease is dominated by changes in behavior and cognition [1]. Currently, there is no cure for VWM. Treatment includes supportive care and avoidance of provocative stressors, such as head trauma and fever [6].

Over the past decade, increased understanding of pathophysiological mechanisms has provided insight into opportunities for therapy. A deregulated integrated stress response (ISR) is the driving pathomechanism of VWM [7]. Modulation of the deregulated ISR improves the phenotype of VWM mice [7, 8]. The ISR can be targeted on several levels. Numerous compounds affecting the ISR have been identified: compounds reducing endoplasmic reticulum stress (chaperones, e.g. ursodiol), modulating eIF2α phosphorylation, modulating eIF2B phosphorylation (GSK3β inhibitors e.g. trazodone and lithium), activating eIF2B (ISRIB, 2BAct and other eIF2B activators) [7, 9], targeting GADD34 (guanabenz, sephin1, salubrinal) [7, 10], inhibiting ATF4, and modulating factors downstream of ATF4 [3]. This means that there are multiple drugs with strong therapeutic potential for VWM.

The year 2021 marked the first therapeutic trial in VWM, an open label phase 2 drug repurposing trial to investigate Guanabenz [8, 11, 12]. Several novel drugs targeting the ISR are under clinical development for neurological indications. Some compounds are currently tested or about to be tested in Amyotrophic Lateral Sclerosis [13, 14]. Such compounds are also of interest in VWM. With emerging new treatments, the field of VWM faces new research and regulatory challenges.

The international VWM registry [3] with world-wide data collection over 20 years currently comprises over 400 genetically confirmed VWM patients, of whom approximately 250 are alive. Dealing with extremely limited patient numbers, a highly heterogeneous patient population, a complex disease course with chronic as well as relapsing–remitting decline, with soon irreversible brain damage, makes drug development challenging. An additional complication is that no validated biomarkers are available that correlate with disease progression. The problematic scarcity of eligible trial candidates will be further worsened if simultaneous trials compete for patients. These issues make classical randomized clinical trials (RCT) virtually impossible in VWM.

To accelerate progress in VWM therapy trial development and delivery, the Vanishing White Matter Consortium (VWM Consortium, www.vwmconsortium.org) was founded, an academia-led collaboration of international VWM experts [3]. The VWM Consortium has published on trial development in terms of trial design, definition of more homogeneous clinical subtypes and phenotype-adapted outcome measures [3]. In particular, to optimize trial efficiency in view of the low number of patients eligible for trials, and to minimize patients on placebo in view of the high unmet medical need, the VWM Consortium felt that placebo control data must be shared [3]. In the current paper, we develop a core protocol, functioning as a template for trials in VWM, facilitating sharing of control data, while allowing flexibility regarding trial details.

### Innovative trial design

Over the past decade, growing attention has been paid to innovative trial design. Various alternatives to traditional RCTs, such as basket, umbrella, and platform trials, are well established in oncological diseases and, more recently, COVID-19 [15–17]. Such designs enable testing multiple investigational medicinal products (IMPs) or multiple conditions for a single IMP simultaneously, in order to improve trial efficiency and enhance drug development [18, 19]. Basket trials are designed to test an IMP in different conditions or disease subtypes [17, 20]. Umbrella trials are used to study multiple IMPs in a single condition [17, 20]. Platform trials include features of both basket and umbrella trials and can be used to investigate multiple IMPs in multiple diseases, disease stages, or disease subtypes [21]. The master protocols of innovative trial set-ups can be adaptive and, for example, the arm with the highest benefits may gain priority in randomization [15, 22, 23].

Regulatory agencies have expressed interest in master protocols. The Food and Drug Administration (FDA) published a guideline on master trial protocols for oncological diseases [18] and is co-founder of the Clinical Trials Transformation Initiative (CTTI) [24]. In the recently revised ‘Guideline on the clinical evaluation of anticancer medicinal products’, the European Medicines Agency (EMA) advises for the first time on master protocols [25]. Further, the European Heads of Medicines Agencies launched a working group ‘Clinical Trial Facilitation and Coordination Group’ [26].

All trial set-ups with a master protocol presume predetermined IMPs, conditions, or biomarkers at the start of the trial [27], although amendments with new trial arms with new IMPs and new diseases are possible. The master protocols contain information on the IMPs and conditions investigated and are usually submitted and registered as a single clinical trial. Currently, guidelines for designing and evaluating master trial protocols are mostly focused on cancer [18, 25]. However, the oncology field differs from the field of VWM and other rare disorders in several respects. The total numbers of patients are higher and outcome measures are generally more uniform. Examples are survival and relapse-free survival. The organization and logistics of a platform trial with multiple sponsors is challenging [18]. In principle, the set-up is the same for the different sponsors; therefore, the use of a single master protocol increases the number of amendments needed and already complicated trials become even more difficult to manage during execution [28].

We suggest the use of a limited core protocol as an innovative trial design for orphan drug development in VWM, facilitating pooled control data as key gain, while at the same time allowing flexibility in trial details and operational efficiency for sponsors. This study presents the design and implementation of a core protocol for clinical trials in VWM.

### Rationale

**Fig. 1:**
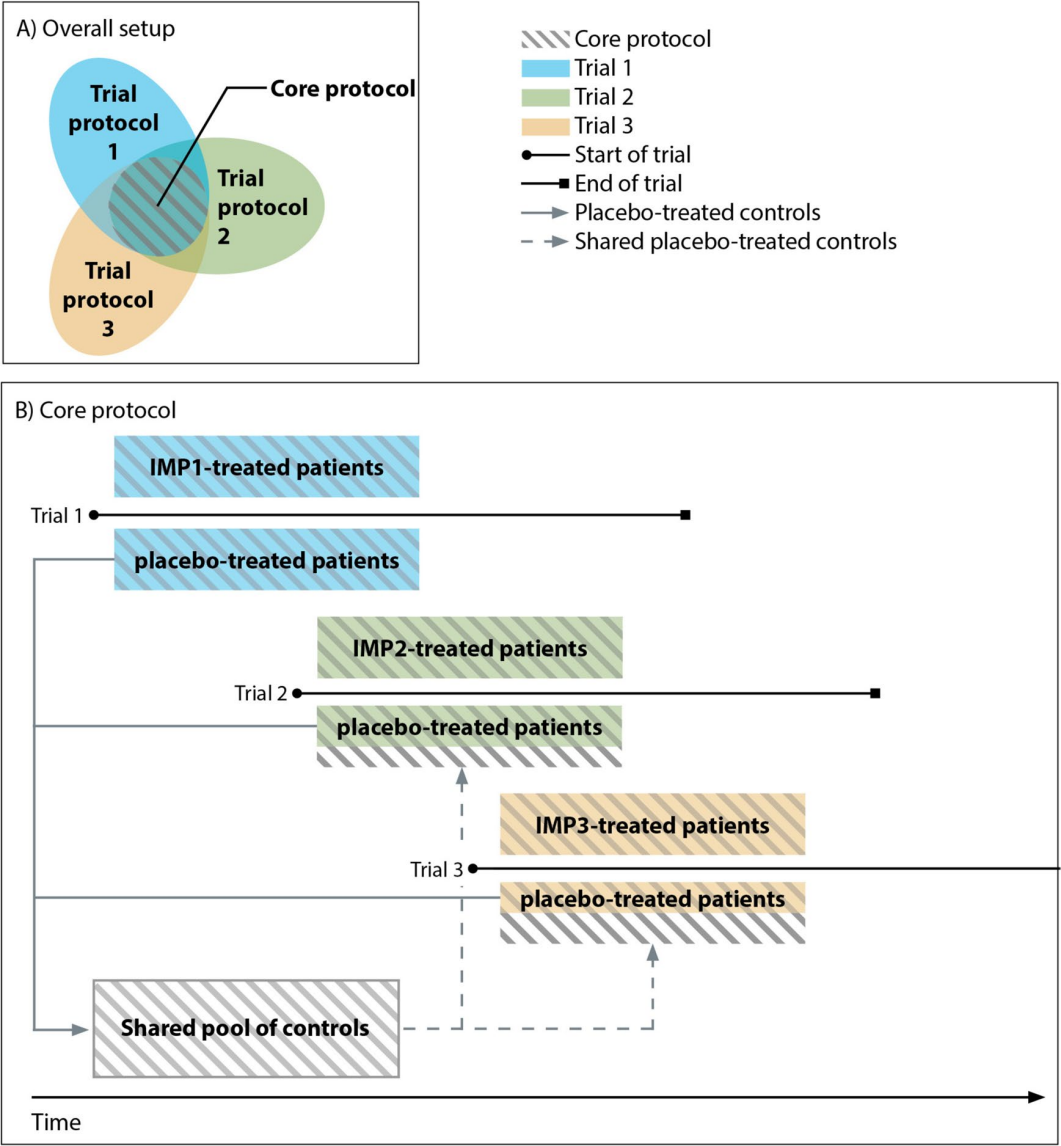
Schematic overview of the core protocol. **A** Venn diagram to show that the core protocol is the common template across all trial protocols. **B** Fictitious impression of parallel and consecutive ongoing trials using the core protocol and contributing to a shared pool of controls. The shared pool of controls, reduces the total number of patients needed to be randomized to placebo. Abbreviations: IMP = investigational medicinal product

The core protocol is designed as a template for phase 2/3 clinical trials in VWM with the purpose to collect safety, tolerability, and efficacy data for marketing authorization (including conditional approval, exceptional circumstances, and accelerated approval) and economic evaluation and benefit-risk assessment as part of health technology assessment (HTA). We combine phase 2 and phase 3 to use the low number of eligible trial candidates efficiently. The template establishes core features for separate trials including a description of the study population, age-specific endpoints, inclusion and exclusion criteria, study schedule of assessments, randomization plan, sample size determinations, and a statistical analysis plan. The core protocol comprises a fixed part, to which participating sponsors are obligated to comply. In addition, there is a flexible, unconstrained part, in which other details, such as pharmacokinetics, pharmacodynamics and biomarkers, can be added (Fig. 1A). The sponsor is free to choose the primary outcome measure. Each trial arm is executed by a sponsor with its own Contract Research Organization (CRO). So, the design harmonizes clinical trial execution in VWM, but does not operate as a single multi-arm multi-stage trial, such as a platform trial. The use of this core protocol enhances efficiency in assessing new therapeutic agents in VWM, because uniformity across trials allows the pooling of data from the control arms and comparison of treatments. In this way, the number of new placebo-treated patients needed per trial can be capped and complemented by placebo-treated controls from the shared pool (Fig. 1B).

### Three age-specific protocols

Disease severity, progression, and manifestations in VWM vary widely for different ages and ages of onset [1], preventing development of a suitable single trial protocol for all patients. Definition of more homogeneous subpopulations is a crucial part of the study design. In alignment with the natural history study [1], we made three separate core trial protocols for: (I) adult patients (current age ≥ 18 years) who have predominantly behavioral and cognitive decline, (II) children (current age ≥ 6 to < 18 years) who have predominantly motor manifestations and (III) young children (current age < 6 years) with very rapid and severe neurological decline. Double-blind RCTs are the preferable trial design and ethically acceptable for protocols I and II. However, for protocol III, because of the rapid disease worsening and early development of severe and irreparable white matter damage, open-label. design is preferable [3]. Over time, placebo controls and historical controls can be replaced by controls receiving the first effective therapy.

Core protocols I and II are very similar since motor, behavioral, and cognitive assessments are part of both protocols. Predominant cognitive or motor decline in older and younger patients respectively may impact the choice of primary outcome measure. The only difference between core protocols I and II is in the details of the neuropsychological assessment, which depend on current age.

The reason for choosing current age of 6 to distinguish between protocol II and III can be explained by the natural history [1]. For younger VWM patients, the natural history study demonstrated that the rate of decline is consistently faster for onset below 4 years than for later onset and correlates with the exact age of onset. For onset from 4 years on, the rate of decline is similar for different ages of onset, with the exception of cognitive decline, which is faster for onset ≥ 18 years. However, some patients with age of onset before 4 years have a slower disease course with milder neurological decline and longer survival [1]. Thus, we have established a criterion of mild to moderate neurological handicap at the current age ≥ 6 years to distinguish between the patients with fast regression compared to slower regression. Patients with onset < 4 years but slower regression can thus be eligible for protocol II. Mild to moderate neurological handicap at current age ≥ 6 is therefore the main inclusion criterion for protocol II.

Based on the numbers of known available participants, the consortium recommends to start trial execution in patients ≥ 18 years, followed by patients ≥ 6–18 years. The number of currently living patients aged < 6 years is extremely low and few patients are known to the consortium [1], hampering trial development.

### Trial eligibility

Predicting VWM phenotype based on genotype is only possible to a limited extent [29]. There are many different genotypes and many patients have private gene variants. Certain genotypes are associated with mild or severe phenotypes [5, 30], but there is considerable intrafamilial variation in disease course [1]. Therefore, when patients are diagnosed genetically, but do not have neurological signs, disease onset and course are uncertain. It is then impossible to assess efficacy of a new investigational treatment. Therefore, neurologically pre-symptomatic patients are not included in the trial protocols. White matter abnormalities on MRI can occur years before neurological manifestations and do not count as neurological manifestations. Also, ovarian failure does not count as onset of neurological disease.

Inclusion criteria are determined in terms of clinical functioning to make sure that there is potential to show stabilized disease [3]. It is known from other leukodystrophies that treating in early disease stages is crucial for good outcomes. For example, hematopoietic stem cell transplantation, as used in metachromatic leukodystrophy, X-linked adrenoleukodystrophy, and Krabbe disease, is able to slow or halt disease progression when applied very early in the disease [31]. Previously established inclusion criteria for VWM trials comprise ambulation without or with minimal support and reasonable cognitive function [3]. Perceptual IQ was previously found to be a better predictor of outcome than verbal IQ in X-linked adrenoleukodystrophy [32]. So, perceptual IQ and ambulation are used as inclusion criteria for trial protocols I and II. For the often severely handicapped patients < 6 years of protocol III, assessment of perceptual IQ is difficult and the criterion of ambulation would exclude almost all patients. To facilitate comparison with historical controls, Health Utility Index (HUI) scores, as collected in the natural history study [1], are used as inclusion criterion.

### Statistical considerations

The heterogeneous disease course and rarity of VWM pose statistical challenges. For this proposal, calculations are based on a minimum study duration of 2 years and the assumption that the IMP stops disease progression. To enhance the power, we propose open-label extension after 2 years until the last patient entering the trial has completed the 2 trial years, with a final assessment for all patients at the end; so if inclusion takes 1 year, the first patients have a trial duration of 3 years. We did not include this recommendation in the power calculations. Of course, if improvement occurs, power calculations should be revised and lower patient numbers are needed. Each sponsor using the core protocol will need to determine a separate statistical analysis plan because the planned sample size and randomization ratio depend on the primary outcome chosen and the associated clinically relevant effect.

The relapsing–remitting disease course combined with a chronic deterioration of VWM imposes additional constraints on data analysis. Episodic deteriorations introduce noise in the data, for which analysis should be corrected. One of the solutions is to separate the mean trend from intra-personal variance by increasing the frequency of measurements. It is, however, necessary to cap the total number of measurements to limit the burden on patients. In this core protocol, patients are assessed at least 6 times: at baseline and 3, 6, 12, 18 and 24 months.

To increase the power of a randomized controlled study with concurrent controls, historical controls may be included as prior information. In the sample size calculations presented in the protocol we do not consider this possibility; although we do use historical controls in the single-arm study for young children (current age < 6 years). Given the fact that the core protocol is based on and aligned with the VWM registry, data from this registry can also be used as a source of historical controls to enrich or validate control groups.

#### Sample size and level of power

Considering the very low number of available VWM patients, overall as well as per age group, a classically powered RCT is not considered feasible in VWM. The conventional statistical analysis of a double-blind RCT is based on a power of 80% with two-sided testing, testing, and an alpha of 0.05.^1^ Strategies for substantially reducing sample sizes include setting the power to 60%, replacing two-sided testing with one-sided testing, and increasing alpha to 0.10. Such settings are common in phase 2b trials and are considered acceptable for ultra-rare diseases [33].

For this proposal, historical data on ambulation and HUI scores, collected in the VWM natural history cohort [1], are used for sample size calculations. Single attribute scores, describing one domain of function, range from 1 (best) to 0 (worst). The HUI generic score is calculated based on the scores of all domains and ranges from 1 (best) to -0.5 (death) [1]. To estimate the sample size from historical cohort data, we assume that the HUI scores follow a normal distribution, that HUI scores are linear in the time since the first HUI assessment, and that the intercept and slopes of the linear trends vary across patients.

In VWM patients ≥ 18 years, cognitive decline is the most prominent symptom and therefore used for power calculations. We chose a HUI cognition score of > 0.32 as minimum to match the inclusion criterion IQ ≥ 50. Based on data from the VWM natural history study [1], the annual decline in the single-attribute HUI cognition score in patients with first assessment at age ≥ 18 years and a baseline score > 0.32 was estimated to be 0.03 points with a residual (within-subject) variance of 0.018, a between-subject intercept variance of 0.009, a between-subject slope variance of 0.001, and a between-subject intercept-slope covariance of 0.001. The standard deviation of the sample of estimated individual slopes was obtained by parametric bootstrapping in a setting with 6 HUI assessments, where data were generated under a mixed effects model with random intercept and slope. The standard deviation of the individual slopes was 0.086. Under the assumption that the experimental treatment stops the cognitive decline, the standard 1:1 randomization design with a power of 0.8, two-sided testing, and alpha of 0.05 requires 130 patients per arm to demonstrate a difference in mean 2-year cognitive decline relative to placebo. The total number of 260 for one trial is not feasible and precludes multiple parallel trials. A sample size of 60 patients per arm would suffice to demonstrate a difference in mean 2-year cognitive decline relative to placebo with 60% power assuming one-sided testing at a 5% significance level. If the significance level is increased to 10%, the number of patients per arm decreases to 40. If only 30 patients are available per arm, then 60% power is achieved when the experimental treatment does not only stop the cognitive decline but leads to a small increase in standardized cognition score of 0.005 points per year. A randomization ratio different from 1:1 enables experimental treatment for a larger proportion of patients. For instance, if the randomization ratio in patients with first assessment ≥ 18 years is set at 2:1, the experimental treatment stops cognitive decline, and the significance level is 10%, the power decreases by only 3% at a sample size of 40 per arm.

In VWM patients ≥ 6—< 18 years, motor decline is most prominent and used for power calculations. We used a HUI ambulation score of > 0.16 to match the inclusion criterion of walking ≥ 10 steps without support or with light support of both hands (GMFM-88 item 67). Based on data from the VWM natural history study [1], the average annual decline in the single-attribute HUI ambulation score in patients with first assessment at age ≥ 6—< 18 years was estimated to be 0.029 points with a residual within-subject variance of 0.008, a between-subject intercept variance of 0.059, a between-subject slope variance of 0.001, and between-subject intercept-slope covariance of 0.001. The parametric bootstrap estimate of the standard deviation of the individual slopes was 0.062. Under the assumption that the experimental treatment stops the motor decline, the standard 1:1 randomization design with a power of 0.8, two-sided testing, and alpha of 0.05 requires 73 patients per arm. The total number of number 146 patients is not feasible. A sample size of 34 patients per arm would suffice to demonstrate a difference in mean 2-year motor decline relative to placebo with 60% power assuming one-sided testing at a 5% significance level. If the significance level is increased to 10%, the number of patients per arm decreases to 22 per arm. If the therapy is expected to increase performance on the ambulation score by 0.01 per year, then the number of patients further decreases to 13 per arm. If the randomization ratio in patients with first assessment between 6–18 years is set at 2:1, the significance level is 10% and the experimental treatment stops motor decline, the power decreases by only 3% at a sample size of 22 per arm.

For patients < 6 years of age, a single-arm open-label study using historical controls for comparison has been recommended previously [3]. The best data available for this age group is the HUI generic score [3]. We chose a HUI generic score of > 0 to exclude patients with a very low level of functioning. Based on data from the VWM natural history study [1], the average annual decline in HUI generic score in patients with first assessment < 6 years and a baseline HUI score > 0 is estimated to be -0.054 points with a residual within-subject variance of 0.018, a between-subject intercept variance of 0.086, a between-subject slope variance of 0.002, and between-subject intercept-slope covariance of -0.001. The parametric bootstrap estimate of the standard deviation of the individual slopes was 0.092.

Hypothesis testing is proposed where the null hypothesis is rejected when the HUI score is higher in the experimental group than in an equally sized group of historical matched controls. Possible matching variables are onset age of disease, disease duration and the baseline HUI value. Under the assumptions that exact matching without replacement is possible, that the outcomes of the treated patient and matched control are uncorrelated, and that the experimental treatment stops further decline, a one-armed superiority design with a power of 0.8, one-sided testing, and alpha equal to 0.05 requires 38 patients. This number is not feasible, but if the power is lowered to 60%, a sample size of 23 patients would be required to demonstrate a difference in mean 2-year HUI decline relative to historical controls. If the significance level is also increased to 10%, only 15 patients would be required. Even lower numbers are needed when the therapy improves the HUI score over time. If the therapy results in an annual increase in HUI generic score of 0.01, then only 11 patients with baseline HUI score > 0 are needed to achieve 60% power assuming one-sided testing at a 10% significance. In practice, exact matching on the matching variables is very unlikely and even inexact matching may be challenging because of the limited size of the pool of historical controls. Therefore, matching should be accompanied with bias correction, for instance by performing a regression on the matching discrepancy. If the pool of historical controls is insufficient, stratification rather than matching may be considered as an alternative method, again accompanied with bias correction. A cruder approach, without matching or stratification, is to conduct superiority testing where the null hypothesis is rejected when the average HUI score change in the experimental group is higher than a superiority margin [34]. If the superiority margin is set equal to the 90% upper confidence bound of the average change in HUI score in the VWM natural history study, sample sizes will be very close to those presented above. For instance, if the treatment stops the decline in HUI score, then 14 patients with baseline HUI score > 0 are needed to achieve 60% power assuming one-sided testing at a 10% significance.

The patients eligible for protocol III overlap with those eligible for the currently ongoing Guanabenz trial, which includes still ambulant patients with disease onset < 6 years [11]. The outcome measures are mostly the same. If interim analyses show efficacy of Guanabenz, comparison with historical control data can be changed into comparison with Guanabenz-treated patients.

#### Alternative approaches

Adaptive enrichment design was considered, as it allows the eligibility criteria to be updated during the trial. However, this results in altering inclusion criteria, which is not compatible with the data-sharing goals of the core protocol.

A Bayesian approach, a methodology based on continuous learning in which observed data prompt adaptations in the probabilities in the statistical model [35, 36], has been used in innovative trials to reduce the sample size needed and increase trial efficiency [16, 23, 33]. However, for the core protocol we considered this suboptimal because of limited available prior information on the primary outcome in the control group. When this prior information is lacking, it is not likely that a Bayesian approach will increase power and it is preferable to choose a classical hypothesis testing approach with predefined criteria for null-hypothesis testing [36], at a type I error of 0.10. After multiple trials have been completed, Bayesian methods can be used to inform a new study with the outcomes of historical controls by means of a power prior. There are static and dynamic versions of power priors where dynamic means that the level of borrowing from historical controls depends on the agreement between current and previous trial data [34, 37]. In practice, the value of historical controls is limited by differences between trials in design and operationalization. Hence, the best way of sharing controls is to combine different trials running at the same time using the core protocol, the data of which can be analyzed with both frequentist and Bayesian metrics [38].

### Outcome measures

Key considerations in the selection of outcome measures were that (a) the collection of outcome measures should be practical and trial burden should be minimized to reduce the risk of dropouts and missing data; (b) the set of outcome measures should cover different functional (motor, cognitive and behavioral) domains; and (c) the assessment tools can preferably be used across broad age ranges and different levels of functioning.

Most of the clinical endpoints were previously published and were selected based on a real-time Delphi consensus procedure (Table 1) [3]. However, we identified gaps requiring several additional tests. Again, the consortium used a consensus-based approach for test selection. To assess hand function, we added the 9-hole peg test [39]. Because extensive neuropsychological tests, such as the Wechsler Adult Intelligence Scale (WAIS) and Wechsler Intelligence Scale for children (WISC), are often not feasible in cognitively disabled patients [3], we included only WAIS and WISC subscales to assess perceptual IQ (which also serves as inclusion criterion). Adding the full WAIS and WISC is optional. The Montreal Cognitive Assessment (MoCA) was added as a brief cognitive test frequently used in adults. The Clinical Global Impression (CGI) [40] and the Caregiver Global Impression (CaGI) rating scales [40], developed by the National Institute of Mental Health, were added as standardized assessment to evaluate treatment effects. Finally, the Food and Drug Administration considers the Columbia-Suicide Severity Rating Scale (C-SSRS) [41] obligatory in protocols for patients > 6 years of age [42].

**Table 1:** Protocol overview

PROTOCOL	I	II	III
**Current age**	** ≥ 18 years**	** ≥ 6—< 18 years**	** < 6 years**
**Design**	Double-blind randomized placebo-controlled trial		Open-label trial
**Inclusion criteria**	Males and females ≥ 18 y of age at screening, with a confirmed diagnosis of neurologically symptomatic VWM^a^	Males and females ≥ 6—< 18 y of age at screening, with a confirmed diagnosis of neurologically symptomatic VWM^a^	Males and females < 6 y of age at screening, with a confirmed diagnosis of neurologically symptomatic VWM^a^
	1. WAIS IV: perceptual reasoning index (based on block design, visual puzzles and matrix reasoning) ≥ 50	1. WISC V: visuospatial reasoning (based on block design and visual puzzles) OR fluid reasoning (based on matrix reasoning and figure weights) ≥ 50	1. HUI generic score > 0
	2. Can walk ≥ 10 steps without support or with light support of both hands (must meet GMFM-88, item 67) 3. Being stable^b^		
**Exclusion criteria**	1. Comorbidity with any relevant disease or condition that would impair assessment of disease progression or of treatment effect, based on clinical judgement of an expert in VWM 2. Unable to undergo MRI due to metal-containing implants, such as cochlea implant, neurostimulator or pacemaker 3. Simultaneous participation in another interventional trial 4. Unable or unwilling to comply with all details of the protocol 5. Situation in which adherence to the study medication or follow-up procedures cannot be guaranteed 6. Known allergy or hypersensitivity to the investigational treatment or to any of the other components of the formulation used in this study		
**Study schedule**	• The treatment period is 2 years • All clinical assessments and body fluid biomarkers are assessed at least at 0, 3, 6, 12, 18 and 24 months; MRI and neuropsychological testing at 0, 12 and 24 months • An open-label extension with annual assessments is recommended until the last included patient has been followed for 2 years. At the end of the overall study, all patients will undergo a final assessment if the previous assessment was ≥ 6 months ago • After the trial, all patients should have access to open-label extension, pending review and approval of the DSMB		
**Common endpoints**	**Clinical endpoints**		
	• Health Utilities index 3 [43, 44], including the self-care item of the HUI2, as described [1] • Vineland Adaptive Behavior Scales, 3^rd^ edition (Vineland-3) • Leiter International Performance Scale (LIPS)—third edition [45] • Gross Motor Function Measure (GMFM-88) [46] • Gross motor function classification in MLD (GMFC-MLD) [47] • Expressive language function classification – MLD (ELFC-MLD) [48] • Manual ability classification system (MACS) [49] • Euro-Quality of Life Instrument 5D, 5 levels (EQ-5D-5L) [50] • Pediatric Quality of Life Inventory (PedsQL) [51, 52] • Clinical Global Impression-Severity (CGI-S) [40] • Caregiver Global Impression-Severity (CaGI-S) [40]		
	**Quantitative brain MRI parameters**		
	• 3D T1-weighted and 3D FLAIR imaging, allowing segmentation into normal cerebral white matter, abnormal cerebral white matter (FLAIR hyperintense), rarefied and cystic cerebral white matter (FLAIR hypointense) and CSF [4] • T2-weighted axial imaging • Diffusion Tensor Imaging (DTI)		
	**Biomarkers in body fluids**		
	• Serum neurofilament light chain		
	**Health economic evaluation**		
	• Medical Consumption Questionnaire (iMCQ) [53] • Productivity Cost Questionnaire (iPCQ) [54]		
	**Adverse events and serious adverse events**		
	Adverse events and serious adverse events are collected from the start of study treatment until the end of the study, applying the most recent version of the National Cancer Institute (NCI) Common Terminology Criteria for Adverse Events (CTCAE, version 5.0, 27-Nov-2017), as well as applying a scale to grade the impact of adverse events on daily life		
	**Safety**		
	Each sponsor defines its safety assessments, to be discussed with the VWM consortium. Long-term safety assessment is part of the post-authorization registry-based studies and includes monitoring for increased risks of cancer and potential consequences of a failed stress response		
**Age- specific endpoints**	• 10 m walk test [55] • 9 hole peg test [39] • Columbia-Suicide Severity Rating Scale (C-SSRS) [41]		
	• WAIS IV: perceptual reasoning index (based on block design, visual puzzles and matrix reasoning) ≥ 50 • Montreal Cognitive Assessment (MoCA) 8.1 (executive function, memory, attention, word fluency, orientation, and visuo-constructive function) [56]	• WISC V: visuospatial reasoning (based on block design and visual puzzles) OR fluid reasoning (based on matrix reasoning and figure weights) ≥ 50	

^a^Confirmed diagnosis: genetically proven VWM with 2 pathogenic or likely pathogenic variants in one of the *EIF2B1-5* genes by ACMG criteria and expert opinion; brain MRI compatible with VWM diagnosis and neurologically manifest disease, as assessed by a physician experienced in VWM

^b^Being stable by clinical assessment of VWM expert: no episode of acute decline for ≥ 3 months before trial entry or complete recovery from more recent episode

Time of death is highly dependent on decisions of parents and health care providers and survival was therefore not considered a reliable endpoint. Because of the extreme variability in the stress-provoked episodes of decline, they were also not considered a useful endpoint.

Recently published work in leukodystrophies has revealed the additional value of quantitative biomarkers in the form of serum neurofilament light chain [57], and for VWM specifically quantitative MRI measures [4]. In the current core protocols, the MRI pulse sequences chosen allow segmentation and quantification of normal gray and white matter, rarefied and cystic white matter, and cerebrospinal fluid (CSF) [4]. Serum neurofilament light chain is part of the core protocol, but no other body fluid biomarkers have been included. Lumbar punctures are not part of the core protocol and are considered optional. We recommend that clinical trials should identify non-CSF biomarkers as much as possible to reduce trial burden. In order to facilitate sharing of control data from different trials, adequate serum and plasma (30 ml blood for patients ≥ 6 years) should be collected at all trial visits. The shared controls samples should in part be stored at a central research organization or a laboratory employed by this research organization, so that subsequent trials have the opportunity to compare their biomarker results.

Importantly, the primary, secondary, and exploratory outcome measure(s) are decided by the sponsor. It is optional to define composite outcome measures. It is up to the sponsor whether additional outcome measures are collected, including patient-defined outcomes. The sponsor defines which change in primary outcome measure will be considered significant. Thus, for the sponsors of each trial executed using this core protocol, there is significant remaining flexibility.

## Design and methods

### Input on protocol design

This core protocol is established based on the VWM consortium expertise with input from patient advocates and a trial statistician, as reflected in the authorship of this paper. Patient advocates from the European Leukodystrophy Association, United Leukodystrophy Foundation, Dutch patient organization for inborn errors of metabolism and leukodystrophies (VKS), and VWM Families Foundation reviewed draft versions and attended a virtual meeting, in which the protocol was discussed and decisions on trial details were made. The core protocol is fully endorsed by these patient advocacy groups [58]. Several published guidelines from regulators were used, including the FDA guideline on Master protocols [18], work from the CTTI [24], and Heads of Medicines Agencies’ Clinical Trials Facilitation and Coordination Group [19].

### Inclusion and exclusion criteria

Patients will be assessed for eligibility according to the inclusion and exclusion criteria (Table 1). Patients will be allocated to the age-specific protocol at the time of screening. All patients must have a confirmed diagnosis of manifest VWM defined as: genetically proven VWM with 2 pathogenic or likely pathogenic variants in one of the *EIF2B1-5* genes by American College of Medical Genetics and Genomics (ACMG) criteria and expert opinion; brain MRI compatible with VWM diagnosis, as assessed by a physician experienced in VWM; and neurologically manifest disease, as assessed by a physician experienced in VWM.

Minimum levels of motor and cognitive function are required for inclusion (Table 1). To reduce the possibility of including patients showing improvement after an episode of acute decline, absence of rapid deterioration for at least 3 months before entering the trial or complete recovery from a more recent episode, as assessed by a physician experienced in VWM, is an inclusion criterion for patients ≥ 6 years. For patients < 6 years of age, such criterion would lead to exclusion of all patients with fast progression and cannot be applied. Epileptic seizures not associated with neurological decline are not considered deteriorations.

### Investigational medicinal product

No detailed pre-specified criteria are defined for IMPs using this core protocol. For a new IMP that is candidate for the core protocol, we recommend a strong biological plausibility and significant beneficial effects in a validated relevant preclinical disease model [38]. Prior to use in this core protocol, a novel IMP should have at least one previous phase 1 trial to provide information about safety and safe dosage of the IMP in humans. No pre-specified criteria are established on the study population of the phase 1 study, although reasonable evidence on safety and PK/PD profiles that can be extrapolated to the study population should be available.

### Shared control pool

Regardless of the success of the trial, the sponsor owning the data will ensure that data of the placebo-treated group is accessible by investigators, other sponsors and regulatory bodies, and guarantee long-term availability of the data. The integrity of the shared control pool will be monitored. For each trial, administration mode, dose frequency, and drug-specific inclusion/exclusion criteria will be addressed in the drug/placebo regimen overview. The risk of bias is reduced by the large effect size aimed for (halting disease progression) measured with robust outcome measures.

### Assignment of interventions and randomization

Regarding trials running in parallel, patients and families are free to determine their trial of preference. Additionally, patients and families will be asked permission, conform the ethics and privacy legislations, for sharing and reusing their personal data in a registry and the shared pool of controls. When shared control data become available for protocols I and II, the allocation ratio (treatment or control) for subsequent trials will be adapted, but placebo-controlled trials cannot completely rely on previously collected control data and become open-label trials. They are set up to be randomized and double-blind and therefore need to contain controls, also to minimize the possible impact of drift in phenotype.

### Statistical analysis plan

All planned analyses need to be pre-specified in a statistical analysis plan, including the estimand strategy and the testing procedure that will be used to test primary and secondary outcomes. Appropriate sensitivity analyses need to be defined. Early stopping for futility can be considered. Early stopping for early efficacy is generally discouraged.

### Study schedule

**Fig. 2:**
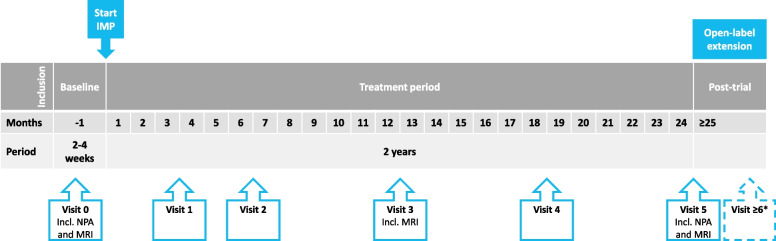
Study schedule*.* * Optional quarterly follow-up until study end or single final assessment at the time of study end. Abbreviations: NPA = neuropsychological assessment

The phase 2/3 trial duration is 2 years. We propose an open-label extension and data collection for all patients until the last included patient has been followed 2 years. The schedule of assessments can be viewed in Table 1 and Fig. 2. A period between inclusion and randomization, known as a run-in period, is not used because (a) it potentially decreases study validity [59], (b) washout periods are not applicable, as the IMP is given next to best supportive care and no other treatments are available, (c) with the variable disease course of VWM a run-in of 3–6 months is too short a time-frame to be sufficiently informative, and (d) patients, especially very young ones, may deteriorate irreversibly in this time frame. An observational period after inclusion is not required because disease stability for the preceding 3 months is an inclusion criterion.

### Safety and quality control

For all trials, a detailed plan for monitoring must be in place. Details on monitoring side effects and safety as well as criteria for stopping the trial should be provided and an independent Clinical Trial Data Safety Monitoring Board (DSMB) must be installed.

### Organizational aspects, data management, and implementation

The sponsor’s CRO is responsible for randomizing patients over the experimental and control arms of the trial and managing the data. Each trial protocol using this core protocol will be separately registered and submitted to regulatory authorities.

After completion of the trial, data of the placebo-treated patients will be shared with a separate research organization arranged in a data transfer agreement between the CRO of the sponsor and the research organization in charge. Once a trial has finished, upon database lock, the research organization will provide the sponsor of the trial with all data of the placebo controls collected in previously completed trials, meaning that the first trial does not benefit from control data collected in other trials. The shared data include outcomes, descriptives and confounders. The trial(s) that are still running will continue to be blinded, as are the clinical trial sites. Through the use of the core protocol, the compatibility and interoperability of the core data elements across the different databases is ensured at the start of each trial. A data dictionary with the database structure and detailed descriptions and coding of the data elements is provided by the research organization and will enable data exchange, even in the presence of different electronic data capture systems.

To encourage the use of the core protocol, the VWM Consortium and patient advocates offer several services to sponsors. The validated VWM mouse model has been made available in an independent CRO, to be used for preclinical testing of the IMP. The VWM Consortium can be consulted to evaluate preclinical data and trial design. The patient registry of the VWM Consortium can be used to recruit patients. The centers represented in the VWM Consortium, currently 8 centers in North America and Europe, are available as study sites. The data collected in the Guanabenz Trial [11] will be made available to the research organization handling the shared control data. Recognizing the value of shared data, the patient advocates are motivated for clinical trial participation with sponsors using the core protocol.

### Post-authorization evaluation

When new products for VWM become commercially available, it is likely that long-term safety and efficacy will still need monitoring. Real-world data collected in a registry can help answering those academic research as well as regulatory and HTA questions [60–62]. In the case of remaining open questions, such as after exceptional circumstances, or conditional or accelerated approval [63], the marketing authorization holder often has the obligation to collect post-marketing data to help answer those open questions. Typically, the marketing authorization holder launches a drug-specific registry or long-term-follow-up study to monitor the authorized drug. We prefer a single academia-led VWM-specific patient registry that can be used for multiple purposes, including academic research and regulatory/HTA decision-making. With this core protocol enabling a shared pool of controls, and the VWM registry, the consortium created favorable conditions for post-launch evidence generation.

## Discussion

VWM is a devastating disease without curative treatment. With increasing insight into disease mechanisms, new therapeutic targets have been identified, and several are now entering the clinical research phase [3]. Given the rarity and heterogeneity of VWM, parallel ongoing trials may further hamper adequate powering of studies. Therefore, together with patient advocates and in open communication with regulators, the VWM consortium has designed a core protocol for registrational phase 2/3 trials in VWM. The resulting uniformity across trials enables building a pool of placebo-treated controls, thereby reducing the number of patients with placebo and the total number of patients needed per trial, and this is crucial [64].

The core protocol presented here is an alternative to platform trials. Prior platform trials [15, 16, 22, 23, 65] have been able to conduct adaptive multi-arm and multi-disease-stage clinical trials [20, 21, 27]. However platform trials designs are highly complicated [28] and, to our knowledge, are challenging to tailor for heterogeneous ultra-rare diseases with a high unmet need. The present protocol was designed to create a straightforward uniform template for parallel and consecutive trials in VWM.

The choice to use a non-randomized trial in patients < 6 years of age with a historical control was difficult. Placebo-controlled trials are standard to demonstrate the causal effects of interventions. However, we believe the rarity, severity, and relative predictability of disease course of VWM at ages < 6 years justify an open-label trial. The VWM registry contains data from approximately 400 patients making it the largest VWM cohort worldwide and can be used to provide historical controls, especially for patients with early onset, which constitutes the largest group. A note of caution is due here, since the natural history itself may be changing. Standards of supportive care may be different for different geographic areas and may change over time. The first complicates international trials with patients from different countries and is a factor difficult to compensate for. Stratification for geography would further reduce the number of eligible trial candidates. The second complicates the use of historical controls. The most obvious change in supportive care is the introduction of preventive measures following identification of the genetic defect in 2001 [66]. For this reason only historical controls diagnosed after 2001 are used.

Other trial designs in which patients serve as their own control, such as personal goal setting and cross-over trials or N-of-1-trials, were considered but deemed inadequate. Personal goal setting was considered unfeasible, because the therapeutic goal is disease stabilization and not improvement of a certain disease sign. On the other hand, there are initiatives to validate goal attainment scaling, an instrument to evaluate change in daily life activities on an individual basis, as an outcome measure in rare disease trials [67]. The disease course in VWM is difficult to predict due to the episodic decline, making cross-over or N-of-1-trials unreliable [68].

An important requirement for the success of this protocol is the commitment of sponsors to comply with the core protocol and share their controls. The sponsors may be motivated to participate by benefitting from the shared pool of controls, the use of the VWM registry, the consortium’s expertise, and the desire of patients and families to participate with “good citizen” sponsors. Clinical drug development in rare diseases remains a complicated and expensive process involving multiple stakeholders. Innovating this process is even more complex. With broad use of this core protocol, the VWM Consortium and patient advocates are optimistic for rapid progress in therapies for VWM.

## Data Availability

The datasets used and/or analyzed during the current study are available from the corresponding author on reasonable request.

## Footnotes

1 Type 1 error is rejecting the null hypothesis that is actually true, whereas type 2 error is failing to reject null hypothesis that is actually false. Probability of type 1 error is known as ‘alpha’ and the probability of type 2 error as ‘beta.’ Power is the probability of making the correct decision/detecting an effect/avoiding type 2 error. Testing can be two-sided meaning that the test evaluates both sides of the spectrum, or one-sided meaning that only in one direction is tested.

## Declarations

### Clinical research standards

All methods were carried out in accordance with relevant guidelines and regulations.
